# Plant interactions can lead to emergent relationships between plant community diversity, productivity and vulnerability to invasion

**DOI:** 10.1038/s41598-024-59996-3

**Published:** 2024-06-17

**Authors:** Elizabeth J. Trevenen, Erik J. Veneklaas, François P. Teste, Mark P. Dobrowolski, Ladislav Mucina, Michael Renton

**Affiliations:** 1 School of Biological Sciences, The University of Western Australia, Perth, Australia; 2 Instituto de Matemática Aplicada de San Luis (IMASL), CONICET, Universidad Nacional de San Luis, San Luis, Argentina; 3 Swift Current Research and Development Centre, Agriculture and Agri-Food Canada (AAFC), Swift Current, SK Canada; 4 Iluka Resources Limited, Perth, Australia; 5 Harry Butler Institute, Murdoch University, Perth, Australia; 6 Department of Geography and Environmental Studies, Stellenbosch University, Stellenbosch, South Africa; 7 School of Agriculture and Environment, The University of Western Australia, Perth, Australia

**Keywords:** Plant ecology, Ecological modelling, Invasive species

## Abstract

Understanding what makes a community vulnerable to invasion is integral to the successful management of invasive species. Our understanding of how characteristics of resident plant interactions, such as the network architecture of interactions, can affect the invasibility of plant communities is limited. Using a simulation model, we tested how successfully a new plant invader established in communities with different network architectures of species interactions. We also investigated whether species interaction networks lead to relationships between invasibility and other community properties also affected by species interaction networks, such as diversity, species dominance, compositional stability and the productivity of the resident community. We found that higher invasibility strongly related with a lower productivity of the resident community. Plant interaction networks influenced diversity and invasibility in ways that led to complex but clear relationships between the two. Heterospecific interactions that increased diversity tended to decrease invasibility. Negative conspecific interactions always increased diversity and invasibility, but increased invasibility more when they increased diversity less. This study provides new theoretical insights into the effects of plant interaction networks on community invasibility and relationships between diversity and invasibility. Combined with increasing empirical evidence, these insights could have useful implications for the management of invasive plant species.

## Introduction

Understanding and predicting invasion in plant communities is a critical question in ecology. Significant progress has been made in identifying traits of invading plants and of resident plant communities that affect invasion success. Invader traits shown to increase invader success (invasiveness) include long-range seed dispersal^1^, the ability to alter growth conditions favoured by resident species^2–4^, and rapid evolutionary change that results in increased competitive ability^5^, such as the evolution of novel traits or novel biochemical ‘weapons’^6^. Resident community traits shown to affect invasion success (invasibility) include niche space availability and the presence of enemies or competitors^7^. While theories suggest that diverse communities may be less invadable due to having fewer available niche spaces and more enemies/competitors^5^, a general relationship between diversity and invasibility is debated^8–10^. Plant-plant interactions can affect species coexistence including the coexistence of competitors, through for example, facilitation^11^. Therefore, plant-plant interactions likely also affect community invasibility^11–13^. However, few studies have explored how plant interactions that affect community traits such as diversity and the presence of competitors affect community invasibility, as it is empirically challenging to do so.

In plant communities, a range of positive and negative interactions between species occur, directly through facilitation or competition, or indirectly through changes to abiotic /biotic conditions. A network approach is increasingly being used in ecology as a framework to explore the complexity of species interactions and their effects on community structure and functioning^14,15^. As collecting empirical data on complete ecological interaction networks is challenging, most studies model invasion in simulated communities with defined interaction networks. This allows for the roles of different factors in the invasion process to be disentangled. Such work has led to the identification of several network properties that can affect community invasibility. These include connectivity—the number of interactions among species^16^, interaction symmetry—the differences in the number or strength of interactions among species^17^, and the network architecture type—the arrangement of interactions among species also known as network topology or structure^18,19^. From such studies, nested architectures, where a group of highly interactive species interact with many other species, with few to no interactions occurring among the other species, have been found to increase invasibility in trophic communities (negative interactions among species)^18^, but not in plant-pollinator communities (positive interactions among species)^19^. Modularity, where species interact in groups/modules, with relatively few interactions occurring among the different modules, has been found to increase invasibility in both trophic and plant-pollinator networks^18,19^.

While studies have explored the effect of network architecture on plant community diversity and resilience^20–25^, few have considered its effects on invasibility^17,21, 26^. Mack et al.^21^ compared the invasibility of plant communities with either intransitive interaction networks or with only negative conspecific interactions, which are both interactions shown to increase species coexistence/diversity^27^. Intransitive interaction networks are where there is no clear “winner” among the competitors. It can be a type of negative ring network architecture where species A negatively affects species B which then negatively affects species C which then negatively affects species A, creating a ring architecture A > B > C > A. Negative conspecific interactions are a type of negative frequency-dependent feedback where individuals of the same species (conspecifics) make conditions less suitable for each other. Mack et al.^21^ found that communities with intransitive interaction networks were less susceptible to invasion than communities with negative conspecific interactions. Kinlock & Munch,^26^ found that intransitive interaction networks were more invadable that transitive networks, where there is a clear hierarchy among the competitors. However, intransitive communities became the least invadable of the three when species richness was low, suggesting invasibility is affected by a complex interaction between network architecture and diversity. As other network architectures of positive and negative interactions likely exist in plant communities (e.g., modular architectures) and can affect species coexistence and diversity^24^, the effects of other architectures on plant community invasibility should also be explored.

It is suggested that how diversity affects invasibility depends critically on the processes regulating diversity in resident communities^12,26, 28, 29^. The same could be true for other lesser studied emergent properties such as compositional stability and productivity, which like diversity, will also affect the presence/strength of competitors^24,26^. As networks of plant interactions can affect emergent community properties, such as diversity and compositional stability, considering their effects on invasibility may help explain relationships between these emergent properties and plant community invasibility.

In this study, we explored how nested, modular, and intransitive plant-plant interaction networks with either positive or negative interactions among species, and with/without negative conspecific interactions, affect community invasibility using a stochastic grid-based simulation model of plant community dynamics.

Specifically, we aimed to investigate:

1. How different network architectures of heterospecific interactions, with and without negative conspecific interactions, affect a plant community’s vulnerability to invasion; and
2. Whether species interaction networks can lead to and explain relationships between invasibility and other emergent community properties also affected by plant species interaction networks, such as pre-invasion alpha diversity, productivity, and compositional stability.

## Methods

### Model overview

To explore how plant interaction networks affect community invasibility, we used a stochastic grid-based simulation model of plant community dynamics, based on a model previously developed by Teste et al.^30^ and later adapted by Trevenen et al.^24,25^. All simulations were performed in R version 4.0.3. The model simulates plant community dynamics on a 100 by 100 square grid, with each grid cell being occupied by at most one individual plant.

Plants interact via plant-soil feedback, a process whereby plants alter the biotic and/or abiotic properties of the soil they grow in, which in turn alters the growth performance of plants growing in that soil^31^. At the start of a simulation all cells are set to be empty, and the soil in all cells is set to be unconditioned. Then, at each time step for the duration of the simulation, the following processes occur in order: immigration, recruitment, growth and mortality. With immigration, each empty cell has a 0.001 probability of being colonized by a new individual chosen randomly from a pool of 100 possible ‘species’, with each species being equiprobable. With recruitment, empty cells are colonised by new individuals that are the offspring of plants currently in the simulated area, with the species identity of these new individuals chosen randomly with a probability weighted by the total current biomass of that species (based on an assumption that larger plants are more fecund). For the first time step, there was no recruitment; this allowed new immigrants from time step 1 and 2 to establish. Dispersal was set to be global, meaning that there was no weighting given to distance from existing plants when choosing the species identity of recruits.

The model represents plant-soil feedback interactions (PSFI) where each plant alters the ‘soil’ properties of its cell, which in turn can either have a positive, negative or no effect on the growth rate of the future occupant of that cell. The size of individual plants is constrained in a simple way by capping the size of neighbouring plants (immediate neighbours so five individuals in total) to not exceed a certain combined biomass. With mortality, each individual has a 0.1 probability of dying and its cell being set to empty (including newly immigrated or recruited individuals). We assume that all species are identical apart from their defined heterospecific interactions; this approach is not intended to represent any real plant community, but it allows us to identify and isolate the effects of plant interactions that are known to exist in such communities. For more details on the model and all these processes see Teste et al.^30^ and Trevenen et al.^24^.

In contrast to Trevenen et al.^24^, we did not explicitly consider current-neighbour interactions as an alternative to previous-occupant interactions in this study. As both Teste et al.^30^ and Trevenen et al.^24^ found that changing the effect type from current-neighbour to the previous-occupant had little effect on patterns of diversity over time, we therefore assume that our study represents plant interactions more generally.

### Plant-soil feedback interaction scenarios

**Figure 1:**
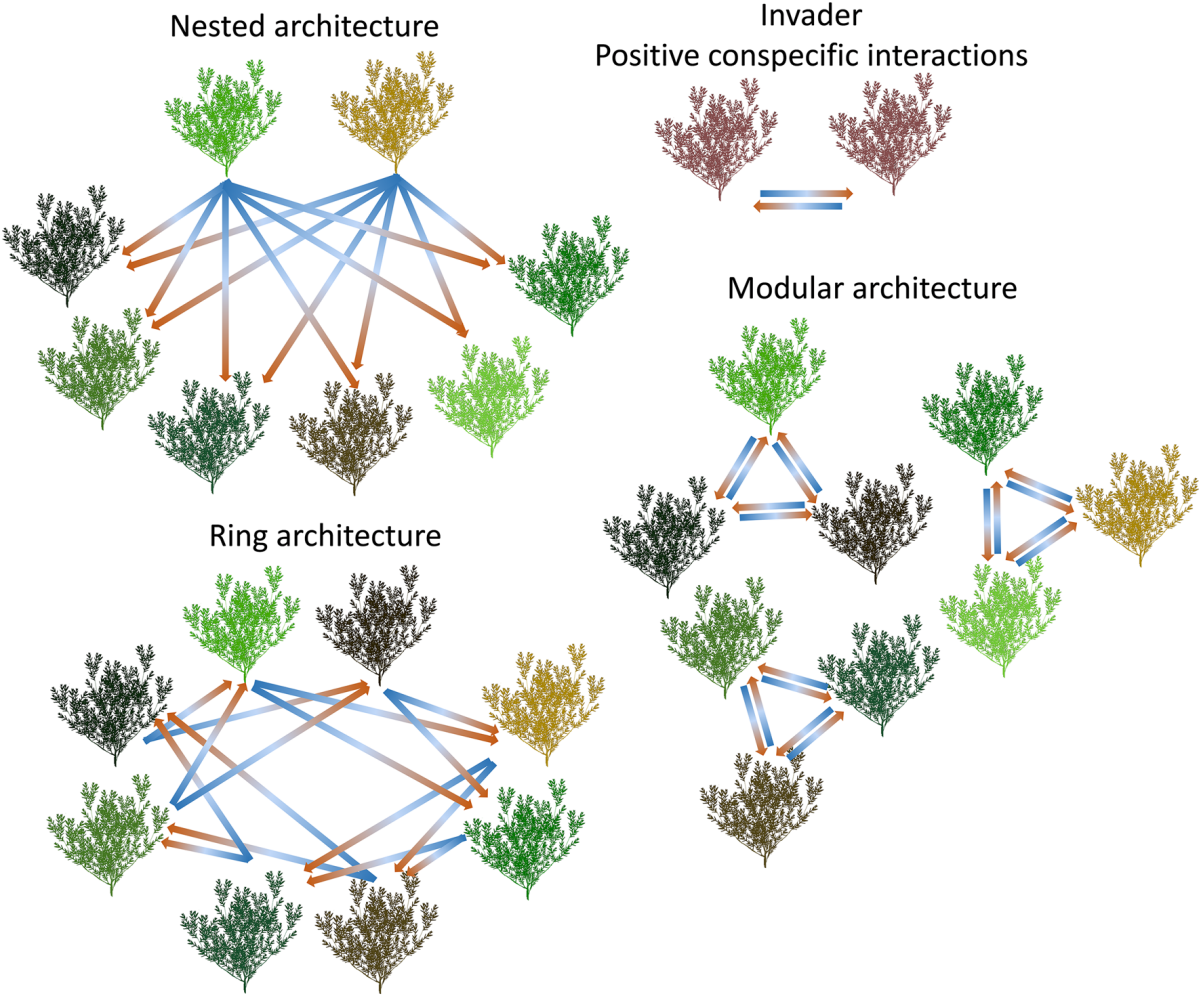
Simplified illustrations of some of the network architectures considered in this study. Different species are represented by shrubs of different colour shades. Coloured arrows represent an interaction (positive or negative) with a blue to orange colour gradient and an arrowhead representing the direction of the interaction. Note that the number of species and thus interactions in our study was much greater than those illustrated in these simplified illustrations, and that the networks in our study are closed systems.

Plant-soil feedback interaction (PSFI) network architectural networks considered in this study included modular, nested, and intransitive ring heterospecific interactions as seen in Trevenen et al.^24^ (Fig. 1). For the nested scenarios, the interactions between species were one-directional, where a number of highly interactive species either positively or negatively affect the growth of all the other species. For modular scenarios, interactions between species occurred in discrete modules of species, where a species either positively or negatively affects the growth of other species within the module, and its growth is also equally affected by the other species in the module. For intransitive ring scenarios (henceforth, ring scenarios), interactions between species were one-directional, where the first species affects a number of other species, each of which in turn affects a number of other species, which in turn affect a number of still different species, and so on, eventually creating an intransitive ring structure.

Several variations of the modular, nested and ring architectural networks were tested. This involved changing the number of species that interacted in each module for the modular networks from 5 to 10 to 20, changing the number of highly interactive species for nested networks from 5 to 10 to 20, and for ring scenarios changing the number of species affected by any given species from 5 to 10 to 20. This variation changed the connectivity of the networks i.e. the total number of interactions among species. For all architectures, we use the term “group size” for this varying number (5, 10 or 20). Note that the number of other species that facilitated/hindered any given species is one less than group size in a modular system, equal to group size in a ring system and either one less than group size (for highly connected species) or equal to group size (for species with fewer connections) in a nested system. This resulted in three different variations of modular, ring and nested PSFI network scenarios. For all these variations, both positive and negative heterospecific PSFI were considered. A negative/positive heterospecific PSFI between species A and species B meant that the growth rate of species A in a soil conditioned by species B was set to be slow/fast. A null scenario (no heterospecific interactions) was also included as a ‘control’, bringing the total number of heterospecific PSFI scenarios considered to (3 + 3 + 3) × 2 + 1 = 19 (Table 1). For each of the 19 scenarios described above, we considered two possibilities for conspecific interactions: the growth rate of species in soil conditioned by a conspecific plant (same species) was either set to standard or to slow (negative conspecific interaction). This resulted in 19 × 2 = 38 different PSFI scenarios in total (Table 1).

**Table 1:** The 38 PSFI scenarios used in this study with details on the heterospecific interaction type (negative implies growth rates of species are slow (slow = 1.49 time step^−1^) in heterospecific soil, positive implies growth rates of species are fast (fast = 1.82 time step^−1^) in heterospecific soil, otherwise heterospecific growth rate is equal to the standard growth rate (standard = 1.65 time step^−1^); group size (changing the number of species that interacted in each module for the modular networks, changing the number of highly interactive species for nested networks, changing the number of species that interacted with other species for ring scenarios); and conspecific interactions (“None” implies growth rates of species are standard in conspecific soil, “Negative” implies growth rates of species are slow in conspecific soil and are indicated by adding NC suffix onto the PSFI scenario name). Information on heterospecific network scenarios with and without negative conspecific interactions are combined in the same row, and separated by a “/”, with information on the scenario without conspecific interactions presented first.

PSFI scenario	Heterospecific interaction type	Heterospecific interaction architecture	Group size*	Conspecific interaction
Null /NC	–	–	–	None/negative
Pos.Mod.5 /NC	Positive	Modular	5	None/negative
Pos.Mod.10 /NC	Positive	Modular	10	None/negative
Pos.Mod.20 /NC	Positive	Modular	20	None/negative
Neg.Mod.5 /NC	Negative	Modular	5	None/negative
Neg.Mod.10 /NC	Negative	Modular	10	None/negative
Neg.Mod.20 /NC	Negative	Modular	20	None/negative
Pos.Ring.5 /NC	Positive	Ring	5	None/negative
Pos.Ring.10 /NC	Positive	Ring	10	None/negative
Pos.Ring.20 /NC	Positive	Ring	20	None/negative
Neg.Ring.5 /NC	Negative	Ring	5	None/negative
Neg.Ring.10 /NC	Negative	Ring	10	None/negative
Neg.Ring.20 /NC	Negative	Ring	20	None/negative
Pos.Nest.5 /NC	Positive	Nested	5	None/negative
Pos.Nest.10 /NC	Positive	Nested	10	None/negative
Pos.Nest.20 /NC	Positive	Nested	20	None/negative
Neg.Nest.5 /NC	Negative	Nested	5	None/negative
Neg.Nest.10 /NC	Negative	Nested	10	None/negative
Neg.Nest.20 /NC	Negative	Nested	20	None/negative

The single invader species was assumed to have positive conspecific interactions, as it is a commonly observed trait amongst successful invading species^32^. Although in nature the invader will likely interact with the resident community, for simplicity, and as countless possible heterospecific interactions between the invader and the resident species could exist, we choose to give the invader no heterospecific interactions. The invader’s growth could still be affected by competition from larger neighbouring plants, as is the case for all resident species. As the invader has the same functional traits as species in the resident community e.g. growth potential, dispersal limitations, and doesn’t interact with the community, our study therefore explores how resident species interactions affect emergent community properties in space and time that then can affect invasibility.

### Simulations and data analysis

Each of the 38 PSFI scenarios was simulated with and without an invasion event, each of these 76 different simulation scenarios was replicated 100 times, and each of these 7600 replicate simulations was run for 12 000 time steps. The 100 replicates were used to account for the variation arising from the stochasticity of the model. 12 000 time steps were chosen to allow the communities to settle into long-term patterns pre-invasion (first 10 000 time steps) and to allow long-term patterns following invasion to be identified (following 2 000 time steps). For simulations with invasion, 100 invader individuals were placed into 100 randomly selected empty cells in the 100 × 100 grid at time step 10 001. Invasibility was quantified as the percentage biomass of the invader in the community at the final time step. To illustrate differences in invasibility among the PSFI scenarios we plotted the average percentage total biomass of the invader with 95% confidence intervals for 2 000 time steps following invasion.

We also considered how the effect of plant interaction network architectures on invasibility relates to other attributes of the resident community also affected by PSFIs, such as diversity, compositional change, and productivity (the average growth rate of the resident community) before invasion. We quantified pre-invasion compositional change using the Bray–Curtis distance, calculated based on total species biomass, to assess the difference in community composition between time steps 5,000 and 10,000. This approach, as recommended by Legendre (2019)^33^, enables us to measure shifts in community composition over time. A higher Bray–Curtis distance signifies significant species turnover or substantial changes in species abundance, indicating increased compositional instability. Pre-invasion diversity was calculated as the Inverse Simpson’s Diversity (1/D) of each community immediately before invasion (at time step 10 000). The average growth rate of the resident community prior to invasion was estimated based on the frequency of each species in the community at timepoint 10,000. This could be considered a measure of the productivity of the pre-invasion community.

All data were generated and analysed using the R environment for statistical computing^34^ with Inverse Simpson’s Diversity calculated using the vegan package^35^.

## Results

### The effect of networks of PSFI on invasibility

**Figure 2:**
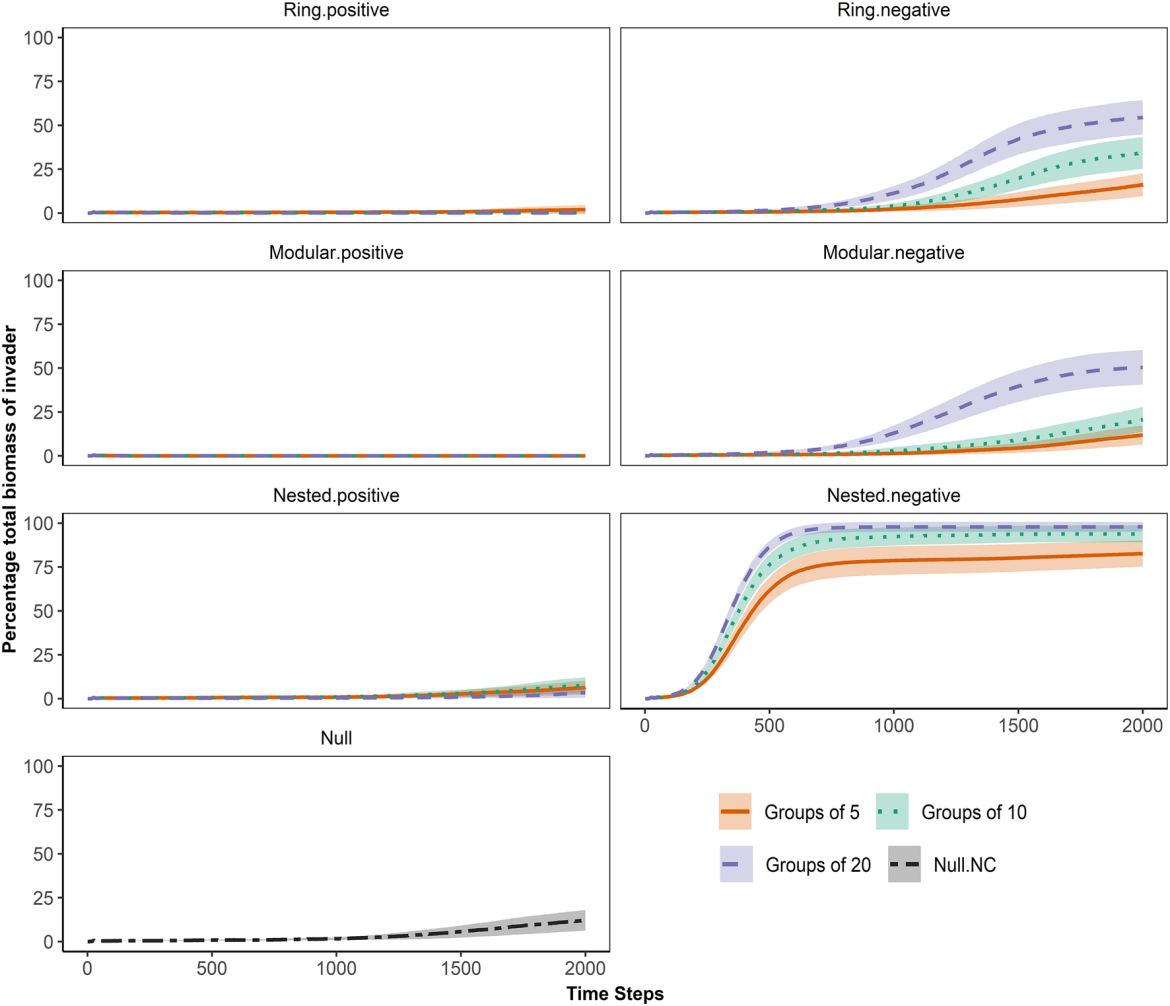
Percentage total biomass of the invader over time in PSFI scenarios with negative conspecific interactions**.** Coloured lines show the average percentage biomass with a 95% confidence interval ribbon calculated from 100 replications. Non-overlapping ribbons at any point in time thus indicates a significant difference between the PSFI scenarios for that time point. Note that confidence interval ribbons are often too narrow to distinguish from the mean line. Different colours represent different group size variations of the networks i.e., number of species in each module for the modular networks; the number of highly interacting species within the community for nested networks; and the number of other species that each species interacts with for ring networks.

Invasibility differed largely among the species interaction network scenarios (Fig. 2). In general, the invader only became dominant (percentage total biomass > 10%) when species in the resident community had negative conspecific interactions as well as negative heterospecific interactions (Table S1). Of those scenarios, the most heavily (and earliest) invaded communities were the negative nested scenarios (Neg.Nest.NC), where the average percentage total biomass of the invader exceeded 80% at the final time step (Fig. 2). The second most invaded communities were those with negative modular (Neg.Mod.NC) and negative ring (Neg.Ring.NC) heterospecific PSFI, in which the average percentage total biomass of the invader varied between ~ 11.5–54% depending on the group size of the network (Fig. 2). For the negative nested, modular and ring scenarios with negative conspecific interactions, the highest levels of invasion occurred when the group size was large (greater connectivity) (Fig. 2).

After scenarios with negative conspecific and negative heterospecific interactions, invasion then occurred most frequently in the null scenario with negative conspecific interactions (Null.NC, ~ 12%), then in positive nested scenarios with negative conspecific interactions (Pos.Nest.NC, ~ 3.5–7.5%). In scenarios with negative heterospecific interactions and no negative conspecific interactions, the average percentage total biomass of the invader ranged from ~ 2.5–4% for negative ring scenarios, ~ 2–2.5% for negative nested scenarios and ~ 0.5–1.8% for negative modular communities. The null scenario with no interactions had similar extents of invasion (Null, ~ 2.2%). Communities with positive ring interactions were only invaded when negative conspecific interactions were present and the group size was small (Pos.Ring.5.NC, ~ 2%). Communities with positive modular interactions were never invaded regardless of the presence of negative conspecific interactions (Table S1).

### Associations among diversity, compositional change and the average growth rate of the community

**Figure 3:**
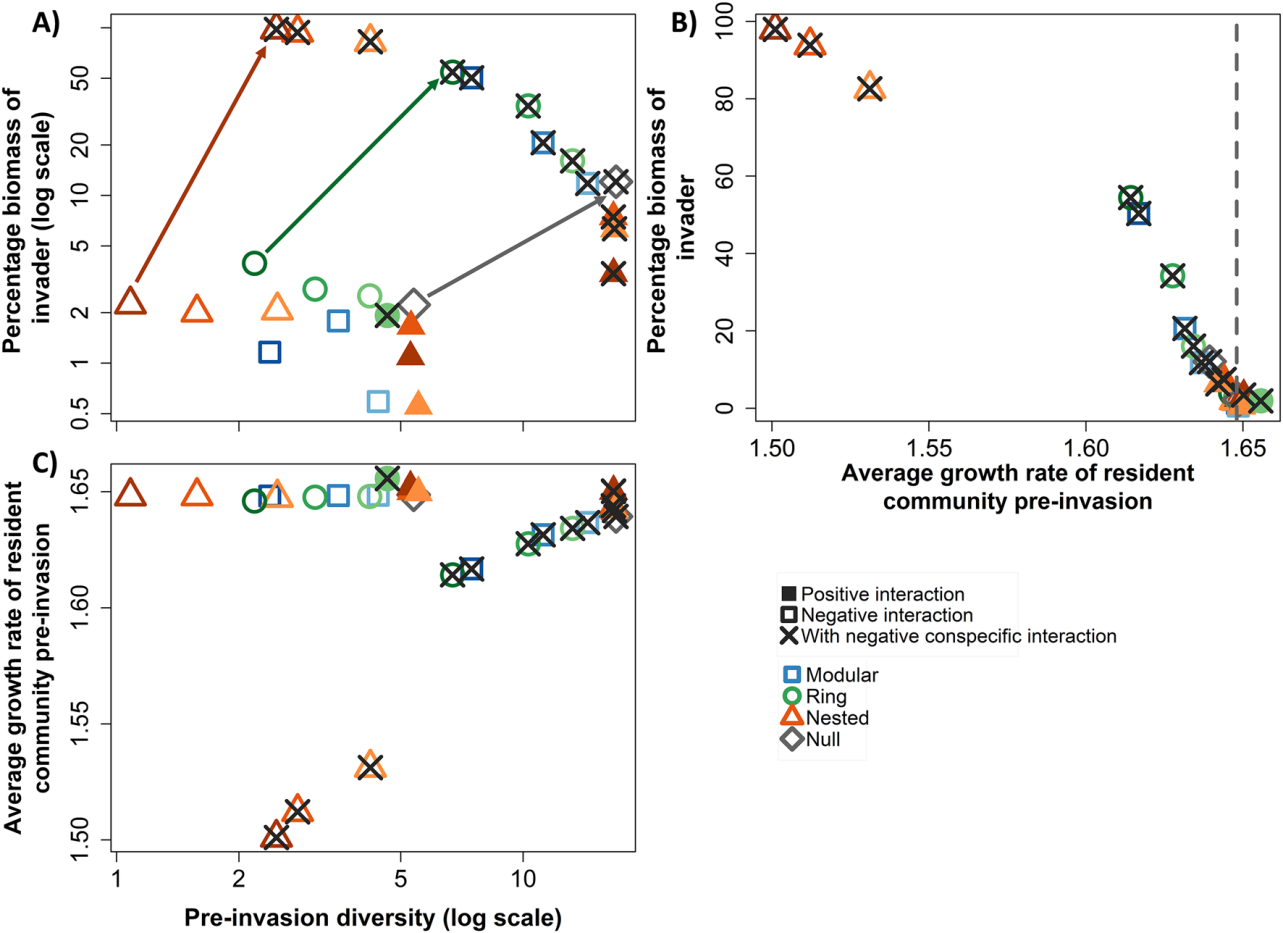
Associations among the average total percentage biomass of the invader at the final time step, and the average pre-invasion diversity (1/D) (**A**), and the average growth rate of the resident community before invasion (**B**). Associations between the average growth rate of the resident community before invasion against the average pre-invasion diversity (1/D) (**C**). Only PSFI scenarios with an average % total biomass of the invader at the final time step > 0.5% are included in these plots. Both axes are logged for plot (**A**) and the x-axis for plot (**C**). Pre-invasion diversity was calculated as the diversity at time step 10,000, just before invasion. The average growth rate of the resident community before invasion was estimated based on the frequency of each species in the community at timepoint 10,000. This could be considered a measure of the productivity of the community. In (**B**) the minimum growth rate of the invader in each community is represented by a grey dotted line. Coloured arrows in (**A**) indicate the changes in values based on the presence of negative conspecific interactions. Symbols represent the means of 100 replicates for each scenario. Different colours and symbols are used to help differentiate the different PSFI network scenarios. Different colour shades represent the different group size variations. Lighter shades represent the architectural variation with a group size of 5, middle shades a group size of 10, and darker shades a group size of 20.

Invasibility strongly related to the average growth rate of the resident community before invasion, with lower average growth rates being associated with a higher invasibility (Fig. 3B). There was also a clear relationship between invasibility and pre-invasion diversity among PSFI scenarios with negative conspecific interactions, with higher diversity being associated with lower invasibility (Fig. 3A).

This relationship was less clear among PSFI scenarios without negative conspecific interactions. However, for each PSFI scenario adding negative conspecific interactions increased both pre-invasion diversity and invasibility (Fig. 3A).

Relationships were also observed between pre-invasion diversity and the average growth rate of the resident community (Fig. 3C). The addition of negative conspecific interactions always decreased the average growth rate of the resident community, yet less so for those with higher pre-invasion diversity.

**Figure 4:**
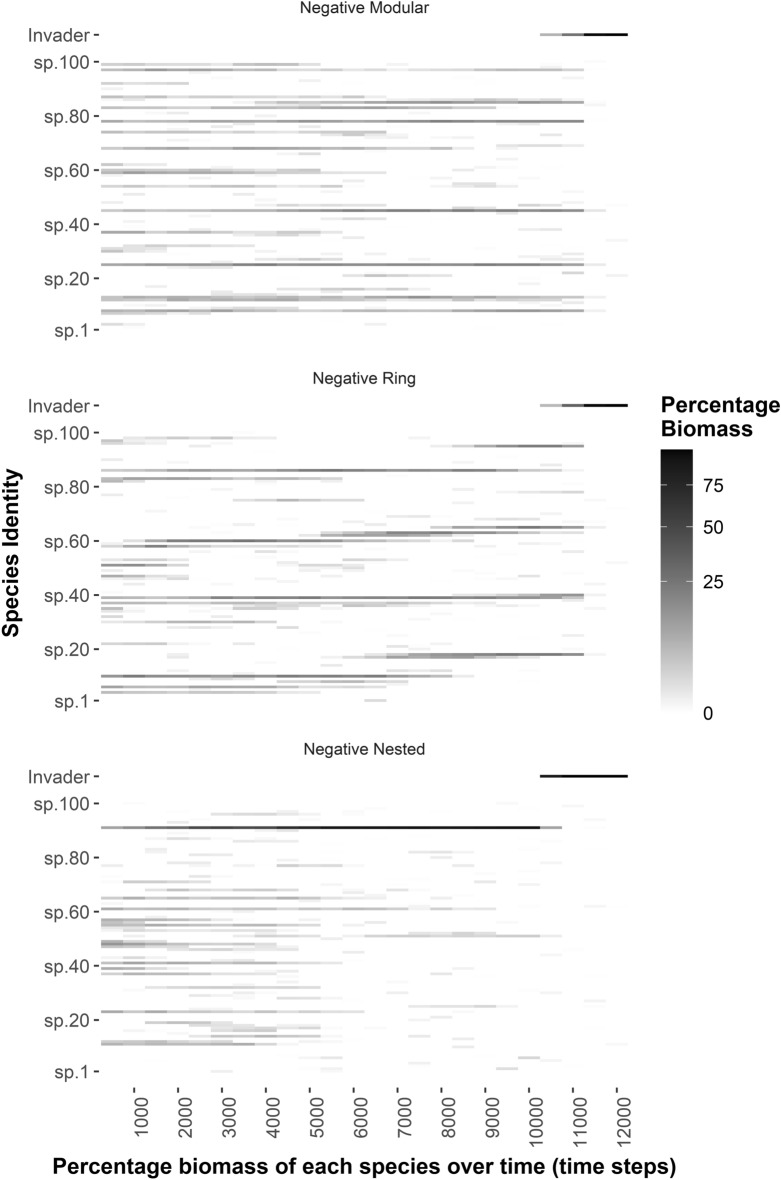
A heatmap displaying the percentage biomass of all 100 species, including the invader species, for a subset of time steps from a replicate simulation for three PSFI network scenarios: Neg.Mod.20.NC “Negative Modular”,” Net.Ring.20.NC “Negative Ring” and Neg.Nest.20.NC “Negative Nested”. Refer to Table 1 for an explanation of these scenarios. These PSFI scenarios are presented because they best help explain our results (see discussion). For the negative nested PSFI network scenario, the 20 species that hinder the growth of the others were species 81–100.

There was no clear relationship between compositional change over time and the average percentage biomass of the invader or the average growth rate of the resident community (Fig. S1) despite large differences in compositional change over time among the scenarios (Fig. 4, S1).

## Discussion

Our results indicate that plant interaction networks can affect a community’s vulnerability to invasion by affecting the average growth rate of the resident community. As a slower average growth rate (productivity) of the resident community equates to a reduced competitive ability, this result is consistent with theory that shows that invasion can occur when the invader has a competitive advantage over resident species^5,6^. However, how plant interaction networks affected the average growth rate of the community, and thus invasibility, was not simply due to the presence/absence of negative or positive interactions. Instead, it also depended on the diversity, dominance, composition, and compositional stability of the communities that result from plant interaction networks.

Our results showed two main diversity-invasibility and diversity-growth rate relationships. Negative conspecific interactions promoted diversity and increased vulnerability to invasion. However, among our PSFI scenarios with negative conspecific interactions, those with higher diversity were less vulnerable to invasion. These apparently contradictory relationships could be explained by considering the mechanism by which plant interaction networks affected diversity and average growth rate. For example, negative conspecific interactions promoted diversity in our plant communities, but also reduced their average growth rate and thus made them more vulnerable to invasion. However, species have a lower probability of propagating into soil conditioned by a conspecific if there is high diversity. Therefore, resident species with negative conspecific interactions are more likely to grow at a faster rate in a diverse community, making them more competitive, and thus these communities are less vulnerable to invasion. Following this, the PSFI scenarios where the addition of negative conspecific interactions did not greatly increase diversity, e.g. negative nested scenarios, had the highest invasibility. It is not surprising that mechanisms that promote species coexistence can also make the community more vulnerable to invasion, as by definition, stable coexistence requires species to be able to successfully invade an established population of the other species^36^. However, interestingly, our results provide a novel explanation for why the relationship between coexistence, diversity, productivity, and community invasibility can be complex, based on plant species interaction networks alone. Therefore, our results add to a growing body of literature that suggests that how diversity affects invasibility depends critically on the processes regulating diversity in resident communities^5,12, 28, 29^.

Our results are consistent with previous work that suggests that a nested architecture makes a community more vulnerable to invasion when the interactions among species are negative^19^. Negative nested interactions can lead to a stable dominance of the competitive “winners” – those that experience the fewest number of negative interactions, even with the addition of negative conspecific interactions which normally reduce species dominance^24^. Our results suggest that in a negative nested scenario with negative conspecific interactions when a highly interactive species dominates, conspecifics, other highly interactive species, and other less interactive neutral species, all grow at a slower rate in soil conditioned by the dominant highly interactive species. As a slower average growth rate of the resident community equates to a reduced competitive ability, this explains why negative nested communities, especially those with negative conspecific interactions, were our most easily invaded PSFI scenario.

Negative modular architectures can lead to one species from each module dominating, resulting in a community with, for example, five non-interacting dominant species for a scenario of five modules^24^. Similarly, negative ring architectures also lead to the dominance of non-interacting species^24^. As the dominant species in these communities were not negatively affecting each other’s growth, the average growth rate of negative ring or modular communities was higher than that of the negative nested communities, thus resulting in ring or modular communities being less invadable. Negative ring communities were slightly more invadable than negative modular communities. This is likely an artefact of the model which meant that more species were negatively affected by soil conditioned by any other species under a ring network architecture compared to a modular network architecture for any given group size (e.g. 20 species under ring scenarios vs 19 species under modular scenarios when group size is 20), thus making the average growth rate lower in the negative ring communities.

Invasion was most prevalent in scenarios with negative heterospecific interactions, but sometimes scenarios with positive heterospecific interactions were also invaded. These included scenarios with positive ring heterospecific interactions and negative conspecific interactions, which have previously been found to encourage extreme species dominance at one point in time^24^. We suspect that under this scenario the population of dominant species in the community temporarily crashes as the probability of propagating in conspecific soil becomes high, resulting in a sharp drop in the average growth rate of the resident community. We believe that this rise of dominance through positive interactions, then crash following the effects of negative conspecific interactions, created a window of opportunity for the invader to establish in these communities.

Positive nested scenarios and the null scenario also saw the establishment and coexistence of the invader. This is because under positive nested interactions, the highly interactive facilitator species become disadvantaged as they experience a fewer number of positive interactions, the reverse of the effect observed for negative nested interaction scenarios explained above. This eventually results in a community containing mostly non-interactive species that is then functionally equivalent to the null scenario (contains only non-interacting species). In these null and positive nested scenarios, the invader’s positive conspecific interactions are enough to allow it to immigrate, coexist and eventually dominate in these communities, especially (but not only) when negative conspecific interactions slow the average growth of the resident community.

Our work is only a first step towards understanding how plant interaction networks can affect the invasibility of communities over time. In many ways our model lacked biological realism. Our approach where species are identical except for differences in species interactions allowed for the effects of plant interaction networks on invasibility to be clearly distinguished, but it also meant that differences among species were relatively subtle which may not be true in nature. Additionally, in nature the invader would likely interact with resident species which could have important implications for invasion as seen in Kinlock & Munch^26^. Moreover, plant interactions resulted in some communities being compositionally unstable over time (Fig. 4). Although compositional stability did not directly relate to invasibility, different compositions of a community could result in different levels of productivity. Therefore, the invasibility of a community that is compositionally unstable may vary over time. Dispersal was also not limited in our model. This meant that we could not explore the effects of species interactions on the spatial clustering and segregations of species across the landscape as seen in other studies^26^ and in nature^37^. With local dispersal, species may be able to avoid interacting with certain other species in the community through spatially distancing themselves from them. As suggested in Kinlock & Munch^26^, this could have important implications for invasibility if spatial patterns due to plant interactions exist despite/on top of the effects of abiotic drivers. Lastly, our 100 species communities were much larger than those used in comparable studies (3–11 species)^21,26^, who found interesting interactions between changes in species number and interaction structure in the effects on invasibility; this also warrants further investigation.

Better application to real systems will require further development of empirical and analytical methods for identifying and quantifying plant species interaction networks. Once species interaction networks can be identified, the long-term effects of interaction networks on community dynamics can then be investigated through simulation studies such as this one. These developments will help the ideas of this study to be applied to real systems.

In conclusion, our model demonstrated that different plant interaction networks can lead to communities with different levels of invasibility. Resident communities with lower average growth rates (lower productivity) were more invadable. The effect of plant interactions on the average growth rate of the resident species was not simply explained by the presence/absence of negative or positive heterospecific interactions, but also depended on the dominance, composition, and compositional stability of the communities as derived from the plant interactions. We also found that plant interactions can lead to and explain apparently contradictory relationships between diversity and invasibility, with negative conspecific interactions leading to a positive relationship between diversity and invasibility and heterospecific interactions leading to a negative relationship between diversity and invasibility. Overall, our results suggest that understanding the mechanisms driving emergent properties such as species coexistence, diversity, productivity and compositional stability, can be useful for understanding complex relationships between emergent community properties and invasibility, and that network architecture of species interactions could be one of these key mechanisms.

### Supplementary Information

Supplementary Information.

## Data Availability

R Code for the simulation model of plant community dynamics used in this study is available through Zenodo 10.5281/zenodo.11064066.
