# Supplementary material for: Plant interactions can lead to emergent relationships between plant community diversity, productivity and vulnerability to invasion
Source: Sci Rep. 2024 Jun 17;14:13932. doi: 10.1038/s41598-024-59996-3 (PMC11183213; doi:10.1038/s41598-024-59996-3)
Supplement: Supplementary file 1 — Supplementary Information. [file 41598_2024_59996_MOESM1_ESM.docx]

## SUPPLEMENTARY MATERIAL

**Plant interactions can lead to emergent relationships between plant community diversity, productivity and vulnerability to invasion**

**Table S1.** The average percentage total biomass of the invader in each of the PSFI scenarios at the final time step (12 000), along with 95% confidence error bound.

| **PSFI scenario** | **Average (%)** | **95% Confidence error bound** |
| --- | --- | --- |
| Neg.Nest.20.NC | 97.94 | 2.67 |
| Neg.Nest.10.NC | 93.85 | 4.77 |
| Neg.Nest.5.NC | 82.59 | 7.48 |
| Neg.Ring.20.NC | 54.45 | 9.75 |
| Neg.Mod.20.NC | 50.36 | 9.82 |
| Neg.Ring.10.NC | 34.24 | 9.08 |
| Neg.Mod.10.NC | 20.63 | 7.11 |
| Neg.Ring.5.NC | 16.09 | 6.51 |
| Null.NC | 12.08 | 5.82 |
| Neg.Mod.5.NC | 11.80 | 5.48 |
| Pos.Nest.10.NC | 7.46 | 4.50 |
| Pos.Nest.5.NC | 6.33 | 3.93 |
| Neg.Ring.20 | 3.94 | 3.01 |
| Pos.Nest.20.NC | 3.42 | 2.85 |
| Neg.Ring.10 | 2.76 | 2.77 |
| Neg.Ring.5 | 2.52 | 2.16 |
| Neg.Nest.20 | 2.25 | 2.20 |
| Null | 2.23 | 1.92 |
| Neg.Nest.5 | 2.07 | 1.64 |
| Neg.Nest.10 | 2.01 | 1.60 |
| Pos.Ring.5.NC | 1.93 | 2.52 |
| Neg.Mod.10 | 1.79 | 1.80 |
| Pos.Nest.10 | 1.66 | 1.59 |
| Neg.Mod.20 | 1.16 | 1.17 |
| Pos.Nest.20 | 1.09 | 1.92 |
| Neg.Mod.5 | 0.59 | 0.33 |
| Pos.Nest.5 | 0.55 | 0.37 |
| Pos.Ring.10.NC | 0.16 | 0.17 |
| Pos.Ring.20.NC | 0.03 | 0.04 |
| Pos.Ring.5 | 0.02 | 0.02 |
| Pos.Mod.5 | 0.01 | 0.01 |
| Pos.Mod.10.NC | 0.00 | 0.00 |
| Pos.Ring.10 | 0.00 | 0.00 |
| Pos.Ring.20 | 0.00 | 0.00 |
| Pos.Mod.5.NC | 0.00 | 0.00 |
| Pos.Mod.20 | 0.00 | 0.00 |
| Pos.Mod.20.NC | 0.00 | 0.00 |
| Pos.Mod.10 | 0.00 | 0.00 |


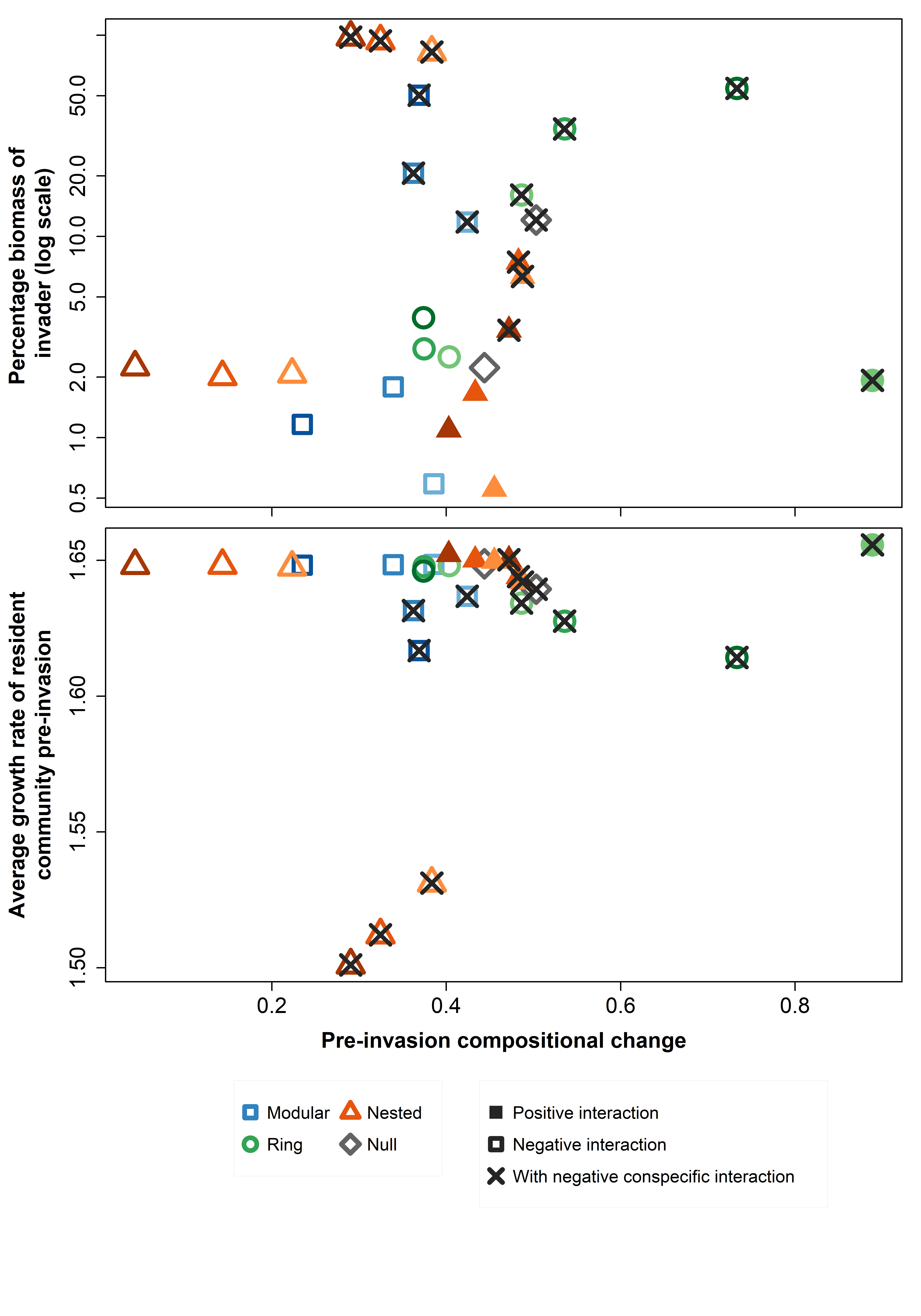


**Figure S1.** Associations between the average pre-invasion compositional change, the average total percentage biomass of the invader at the final time step (12 000), and the average growth rate of the resident community prior to invasion. Only PSFI scenarios with an average percentage total biomass of invader >0.5 % at the final time step are included in these plots. Compositional change was calculated by comparing the Bray Curtis distance between a community at time step 5 000, to the same community at time step 10 000 (prior to invasion). The average growth rate of the resident community prior to invasion was estimated based on the frequency of each species in the community at timepoint 10 000. This could be considered a measure of the productivity of the community. Symbols represent the means of 100 replicates for each scenario. Different colours and symbols are used to help differentiate the different PSFI network scenarios. Different colour shades represent the different group size variations.
